# Economic Performance and Sustainability of a Novel Intercropping System on the North China Plain

**DOI:** 10.1371/journal.pone.0135518

**Published:** 2015-08-14

**Authors:** Chengdong Huang, Quanqing Liu, Nico Heerink, TjeerdJan Stomph, Baoshen Li, Ruili Liu, Hongyan Zhang, Chong Wang, Xiaolin Li, Chaochun Zhang, Wopke van der Werf, Fusuo Zhang

**Affiliations:** 1 Center for Resources, Environment and Food Security, China Agricultural University, Beijing, China; 2 Institute of Agricultural Resources & Environment, Hebei Academy of Agricultural and Forestry Sciences, Shijiazhuang, China; 3 Development Economics Group, Wageningen University, Wageningen, The Netherlands; 4 Centre for Crop Systems Analysis, Wageningen University, Wageningen, The Netherlands; Murdoch University, AUSTRALIA

## Abstract

Double cropping of wheat and maize is common on the North China Plain, but it provides limited income to rural households due to the small farm sizes in the region. Local farmers in Quzhou County have therefore innovated their production system by integration of watermelon as a companion cash crop into the system. We examine the economic performance and sustainability of this novel intercropping system using crop yield data from 2010 to 2012 and farm household survey data collected in 2012. Our results show that the gross margin of the intercropping system exceeded that of the double cropping system by more than 50% in 2012. Labor use in the intercropping system was more than three times that in double cropping. The lower returns per labor hour in intercropping, however, exceeded the average off-farm wage in the region by a significant margin. Nutrient surpluses and irrigation water use are significant larger under the intercropping system. We conclude that the novel wheat-maize/watermelon intercropping system contributes to rural poverty alleviation and household-level food security, by raising farm incomes and generating more employment, but needs further improvement to enhance its sustainability.

## Introduction

Increasing land productivity in agriculture is needed worldwide to sustain growing food demand without claiming more land for agriculture [1]. In China, particularly, there is a great need for high land productivity due to the small arable land area of only 0.09 ha per head of the population [2] and the national policy goal of remaining self-sufficient in grain production [3]. In addition, in China, there is a pressing need for a reduction in the environmental side-effects of high fertilizer use, to combat acidification of farmland and eutrophication of surface water, and mitigate the energy demands of nitrous fertilizer production [3–5]. Therefore the government encourages “double high” technologies that pair high yields with high nutrient use efficiency [6–9].

Farming in China is characterized by small family farms, usually less than one ha, on which farm income is an important element of the livelihood [10]. To deal with the scarce land and comparatively abundant labor resources, rural households use on-farm income diversification, off-farm income diversification or a mixture of both strategies to improve livelihoods. On-farm income diversification usually takes the form of temporally complementary activities. Farmers can, for example, rotate different crops in order to make a fuller use of their land, labor time and other production resources [11].

One option for making fuller use of the available resources is the use of intercropping. Intercropping is the cultivation of two or more crops at the same time in the same field. Crop species may be grown completely mixed, in alternate rows, or in narrow strips of the individual crop species [12]. Intercropping sustains higher land productivity than cultivation of sole crops because mixed species are better able to capture the biophysical resources such as radiation, water and nutrients, due to species complementarity in resource capture patterns in time and space [13]. Intercropping in the 1980s accounted for approximately half the total cereal production in China [14], but nowadays its sown area is probably lower [15], possibly 20–25% of the total sown arable land area [16]. Nevertheless, intercropping is still a vigorous component of today’s agriculture in China, and new systems are continuously being developed in response to changing market prices for labor and agricultural produce and to environmental conditions.

Adding an extra crop to a sole major crop can enhance farmers’ incomes as has been observed elsewhere for banana [17] and potato, tomato or maize [18, 19]. In the North China Plain, the bread basket of China, the local famers, in order to diversify their production system and increase their income, have recently explored new types of intercropping: watermelon/cotton, watermelon/maize, wheat/watermelon in rotation with cabbage or mustard, and wheat-maize/watermelon. Other aspects, like employment generation might also play a role but has not been examined before. Nor has the sustainability received a rigorous analysis so far. Here we focus on the wheat-maize/watermelon intercropping system as a case study. Its main innovation lies in the combination of the traditional wheat-maize double cropping system, which is the dominant cropping system in the North China Plain, with a cash crop that is increasingly being demanded in China’s urban areas. Hence it combines intercropping and double cropping and has the potential to contribute to grain self-sufficiency while improving rural incomes, two major policy goals in China. We conducted crop yield monitoring from 2010 to 2012 and a farm household survey in 2012 to answer the question: What is the economic performance and sustainability of the wheat-maize/watermelon intercropping system given prevailing resource scarcities and the economic and policy environment in the region?

## Materials and Methods

The study was carried out in Houlaoying village, Quzhou County, Hebei province in the North China Plain. Farmers in Houlaoying village recently developed the wheat-maize/watermelon intercropping system. In this study, we compare yields, nutrient budgets, labor use and economic returns of the wheat-maize/watermelon system with those of the traditional wheat-maize double cropping. The wheat, maize and watermelon yield data were collected through crop yield monitoring from 2010 to 2012; the economic returns, labor use and nutrient budget information were collected through farm household surveys held in 2012. In this section we first detail the study site, and then describe the cropping systems and the methods of data collection and data analysis.

### Site description

Quzhou County is located in the center of the North China Plain. Wheat-maize double cropping is the dominant agricultural system. Over the period 2010–2012 the average rural population of the county was almost 406,000 and equaled 90.5% of the total population. Rural per capita net income during the same period was 941 USD yr^-1^ [20]. Quzhou County is home to one of the Science and Technology Backyards of China Agricultural University in which new agricultural technologies, adapted to local conditions, are developed and demonstrated.

**Fig 1:**
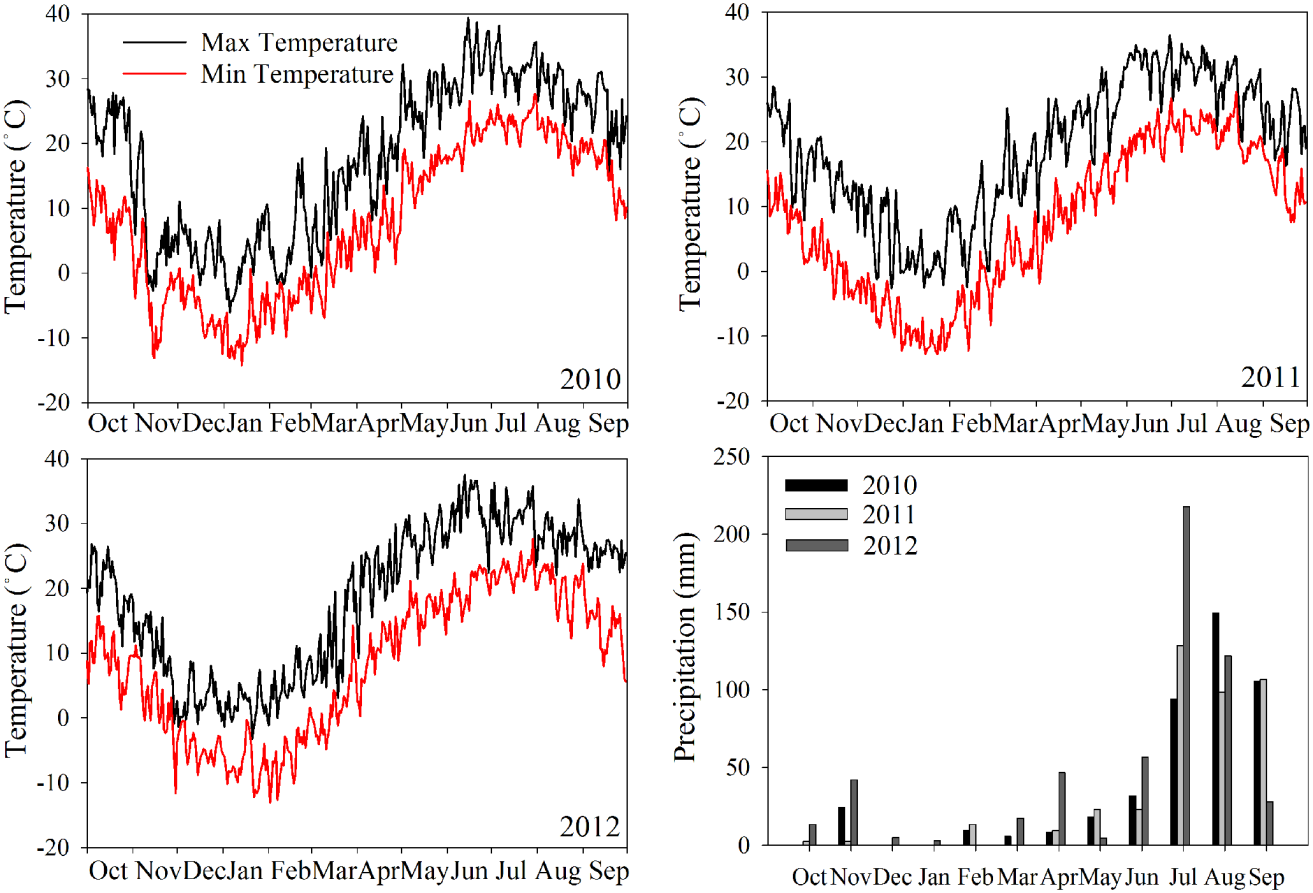
Maximum and minimum temperatures in 2010 to 2012, and precipitation from October–September.

The study site, Houlaoying (36°39′N, 114°55′E and 40 m elevation) is located 20 km southwest of Quzhou County capital. In 2010, the village had a population of 1,211 persons, living in 268 households. A family in Houlaoying had on average 0.58 ha of cultivated land (0.12 ha per capita) of which more than 85% was irrigated [21]. Over the period 2010–2012, the annual average temperature was 13.0°C (Fig 1). The annual rainfall was 480 mm, with approximately 60% concentrated in July and August (Fig 1), and the frost-free period was about 200 days (source: Meteorological station in Quzhou Experimental Station). The soil type is a calcareous fluvo-aquic soil with sandy loam texture and a pH of 8.3. The top soil (0–30 cm) contains 16.0 g organic matter, 0.77 g total N, 24.5 mg Olsen-P, and 158 mg available K per kg air-dried soil.

### Description of cropping systems

Here we first describe the typical features of the two systems that we compare.

#### Wheat-maize double cropping

The wheat-maize double cropping system consists of a winter wheat crop sown in October, and harvested in mid-June of the next year, and a maize crop sown shortly after the wheat harvest and harvested in October. After this, wheat is sown again, and the whole cycle restarts. For brevity, the growing seasons are labeled according to the year when the crops are harvested. The sowing and harvesting of wheat and the sowing of maize are mechanized, while the harvesting of the maize cobs is done by hand. Wheat is sown at a seed rate of 180–300 kg per ha and a 15 cm row distance, and maize is sown to a plant density of 5.6–6.4 plants per square meter at 60 cm row distance. Plant distance within the maize rows is 26–30 cm.

There are 3–4 rounds of flood irrigation during the growth of wheat, with the first before the winter, another at the tillering and/or stem elongation stages, and a final one at flowering. There is one irrigation for maize, immediately after sowing. There are three fertilizer applications in wheat: basal fertilizer before sowing, a first top dressing between tillering and stem elongation, and a last application at flowering. There is a single fertilizer application in maize at sowing. Compound fertilizer is used for basal fertilizer in both crops, while single element fertilizers are used for topdressing. Detailed information about irrigation and fertilization in the system is given in the upper part of Table 1.

**Table 1:** Basic information on irrigation and fertilizer applied to the double cropping and intercropping system.

Cropping system	Irrigation		Fertilization			Nutrients input (kg ha^-1^) ^2^		
	Period	Amount (mm) ^1^	Period	Target Crop	Approach	N	P_2_O_5_	K_2_O
Double cropping (wheat—maize)	Before wintering	120	Before ploughing for wheat	Wheat	Broadcast	150	120	70
	Wheat jointing	120	Wheat Jointing	Wheat	Broadcast	120	0	0
	Wheat flowering	105	n/a ^3^	n/a	n/a	n/a	n/a	n/a
	After maize sowing	130	Maize sowing	Maize	Furrow	120	60	60
	**Total**	**475**	**Total**			**390**	**180**	**130**
Intercropping (wheat—maize/watermelon)	Before wintering	120	Before ploughing for wheat	Wheat	Broadcast	150	120	70
	Wheat Jointing	120	Wheat jointing	Wheat	Broadcast	120	0	0
	Wheat flowering	105	At ploughing for watermelon	Watermelon	Broadcast	115	115	115
	Watermelon transplant	100	n/a ^3^	n/a	n/a	n/a	n/a	n/a
	1st topdressing for watermelon	125	1st topdressing for watermelon	Watermelon	Furrow or spot	115	30	125
	2nd topdressing for watermelon	105	2nd topdressing for watermelon	Watermelon	Fertigation	100	25	110
	Maize jointing	105	Maize jointing	Maize	Furrow or spot	140	0	0
	**Total**	**780**	**Total**			**740**	**290**	**420**

Source: Own fieldwork observations

^1.^ The water use in each irrigation round was derived from the electricity consumption of water pumps. The electricity consumption is recorded with the purpose to charge farmers the cost of irrigation at the end of growing season. Since all farmers use similar irrigation methods, we can roughly estimate water use from information on power consumption.

^2.^ Fertilizer application refers to routine agronomical practices observed among farmers who adopt double cropping and/or intercropping.

^3.^ Fertilization is not applied at wheat flowering stage and watermelon transplanting stage.

#### Wheat-maize/watermelon intercropping system

Wheat-maize/watermelon is a strip-based system. Wheat is typically grown in strips of seven rows at 15 cm row distance. In terms of space occupied, each wheat row covers 15 cm row width, and the strip accordingly occupies 105 cm. Between wheat strips there is an “empty space” equivalent to 5 wheat rows (75 cm). The relative density of wheat in the intercrop as compared to a sole crop is therefore 7/12 = 0.58.

The watermelon is sown in the middle of the empty space between the wheat strips in early May, or transplanted from seed beds in mid-May. Watermelon density is 8,500–10,100 seedlings per ha with a plant distance in the row of 55–65 cm. This planting pattern is consistent with how farmers usually plant sole watermelon. So, the relative density of watermelon equals 1.00.

Wheat is harvested in mid-June and plant residues are returned to the field through the combine harvesters. One to two weeks after wheat harvest, maize is manually planted slightly outward from the position of the outer wheat rows. The inter-row distance of maize is therefore approximately 105 cm across the harvested wheat strip and 75 cm across the watermelon strip. Distance within the maize row is 35–40 cm between seeding holes, with 2 plants per hole. Maize sowing density ranges from 56,000 to 64,000 plants per ha, similar to maize in wheat-maize double cropping. Thus, maize has a relative density of 1.00 in the intercrop as compared to a sole crop. The watermelon plants are not constrained by their “strip space” and grow freely between the maize rows. The watermelons are harvested by hand from mid-July to early August. After the last watermelon harvest, all stems are pulled and left on the field. Maize then grows alone until harvest in early to mid-October.

Comparing the two systems, we observe that the relative density of wheat in intercropping to double cropping is 0.58, and the relative densities of maize and watermelon are both 1.00. More water and nutrients are applied to the wheat-maize/watermelon than to wheat-maize double cropping. There are seven irrigations in this wheat-maize/watermelon system, i.e. four more than in the double cropping. Three of the irrigations are identical in timing and water amounts to those in the wheat-maize system. The extra irrigations are given in mid-May when watermelon is transplanted, mid-June, between wheat harvesting and maize sowing, early July and early August to support fruit production of melon and initial growth of maize (see lower part of Table 1). The irrigations in intercropped watermelon are identical in timing and water amount to those in sole watermelon.

Farmers give the same amount of fertilizer to wheat and maize in the intercrop as they do in double cropping, without a reduction in the fertilizer amount per ha in wheat to account for the bare strips. The basal fertilization before wheat sowing is broadcast over the whole area (wheat strip + watermelon strip), while top dressing of wheat is only applied to wheat strip by broadcasting, again using amounts per ha that are the same as in double cropping (Table 1). There are usually three additional fertilizer applications for watermelon. Basal fertilizer is applied before sowing or planting in the watermelon strip, and ploughed in. The first top dressing is applied after wheat harvest through furrow or individual plant application i.e. at 15–20 cm from the watermelon plants, and the second top dressing is applied during fruit development, simultaneous with full field flood irrigation. After the last watermelon harvest, the final fertilizer application in maize is carried out by placing fertilizer next to the maize rows before irrigation or in conjunction to effective rainfall.

### Data collection

From 2010 to 2012, yields of the two systems were monitored on farmers’ fields that were selected for the study. Oral permissions were obtained from land owners before yield monitoring. In 2012, a household survey was carried out among the selected farmers to obtain information on economic returns, labor use and nutrient budgets of the two systems.

We obtained the household name list from the village group heads and asked them information about ongoing cropping practices. Village group heads in China possess a full name list of the households living in their villages. They also collect information about a range of issues, including planted areas and yields of various crops, which they report to the relevant statistical agencies. Based on the information provided by the village heads and on personal talks with potential participants, we selected 59 farmers who were younger than 70 years old, had an interest in agricultural practices and improving their skills, and were living in the four village groups where the two cropping systems co-exist (see S1 Text for details).

The selection of the participants took place without interference by the village group heads. As the village group heads had no personal interest in the survey, we have no reason to expect that there has been any pressure from the village group heads on farmers to participate. As the focus of our research is on the economic performance and sustainability of an intercropping system, there do not seem to be any controversial or risky issues involved and we did not attempt to identify any vulnerable groups that might be negatively affected by our research. Participants were informed face-to-face about the purpose of the survey and data were recorded only if they were willing to participate. Farmer participation in the study was voluntary. The interviewee’s name was recorded during the survey, but survey data were anonymized before data analysis.

At the time of the survey, no human ethics or data protection committee had been installed at China Agricultural University to which most of the authors, including the first and corresponding author, are affiliated. Nor was approval by such a committee required under the prevailing law. At Wageningen University in the Netherlands, to which the non-Chinese authors are affiliated, scientific research should follow the ‘Netherlands Code of Conduct for Scientific Activities’ [22] and the ‘Code of Conduct on the Protection of Person Data in Scientific Research’ [23]. The methods that were used for collecting and analyzing the survey data for this study are in agreement with these codes of conduct.

#### Yield monitoring

About 60% of the fields with wheat-maize/watermelon intercropping are located in a contiguous wheat-maize/watermelon growing area in the northeast of Houlaoying village, while the remaining plots are scattered around the perimeter of the village. Wheat-maize double cropping plots and wheat-maize/watermelon cropping plots were selected from both the contiguous area and the perimeter of the village. The share of each cropping system in the total sample was equal to the share of the surface covered by that system. The sample sizes varied between systems and years, with a minimum of 9 samples per crop system in each year, and a maximum of 35. Detailed information about the plot sampling and yield monitoring can be found in the S1 Text.

Measurements of grain yield were made at crop maturity. In each sampled wheat and maize field three subsamples were taken. A subsample in wheat consisted of a one meter-long strip of seven adjacent rows, and a subsample in maize consisted of a five meter-long strip of four maize rows. All maize cobs were threshed manually after air drying and the grains were weighed. From each plot a 500-g sample of wheat or maize grain was dried to constant weight in an oven at 70°C, to determine water content of fresh grain. The reported final yields were standardized to 13% moisture for wheat and 14% moisture for maize. Watermelon fruits were picked when mature from late July to mid-August and sold in the free market. The farmers whose fields were used for the study recorded the fresh weight of watermelons collected from the field. The fresh weight was determined using a wagon balance (2 kg to 200 ton operating range). At the end of the watermelon season, the total fresh fruit yield for each plot was calculated.

#### Farm household survey

In 2012, a farm household survey was held among the owners of the plots that were used for the yield monitoring. A total of 59 farm households, responsible for 64 plots of land (29 for double cropping and 35 for intercropping), were interviewed. Questions included in the questionnaire (S1 Table) focused on age, education, land use, agricultural inputs and labor use, and agronomic practices. Agricultural inputs comprised the costs of pesticides, herbicides, seeds or seedlings (watermelon transplants), irrigation water, fertilizers, mulching film (for watermelon seedlings), machinery hire and gasoline for transportation of grain and fruits from the farm to the local market. Labor use was evaluated by asking the number of hours spent on crop and land management activities, including land preparation, mulching, sowing, transplanting, fertilizer application, irrigation, pesticide spraying, weeding, pruning, harvesting, and plant residue processing. Labor use does not include time spent by machine operators. The hours and cost of hiring an operator is included in the rent of agricultural machinery. Basic characteristics of the interviewed farm households are presented in Table 2.

**Table 2:** Characteristics of interviewed farm households.

Cropping system	Interviewed farm households		Population	Laborers			Age distribution of laborers (%)			
	Number (%)	Females		Total (% ^1^)	% Male	Mean age	31–40	41–50	51–60	>60
Double cropping	24 (40.7%)	8	147	49 (33.3%)	53	52.5±9.4 ^2^	14.3	28.6	32.7	24.5
Intercropping	30 (50.8%)	10	164	62 (37.8%)	50	50.0±8.1	16.1	40.3	32.7	11.3
Double cropping & Intercropping	5 (8.5%)	1	18	8 (44.4%)	50	48.0±11.1	0.0	62.5	0.0	37.5

Source: Own farm household survey, 2012

^1.^ Value between brackets is the percentage of laborers in the total population of the households of the cropping system.

^2.^ Values are shown as means ± standard deviations.

Nutrient balances were examined by combining household survey data on plot-specific nutrient input with nutrient output data obtained from grain and fruit samples taken from 40 plots (23 for double cropping and 17 for intercropping). Watermelon nutrient content could only be determined on 17 of the intercropped plots, because of the limited drying facilities available in the village.

Nutrient input includes only mineral fertilizer use in this study. It was calculated by interviewing farmers with a questionnaire (S2 Table) immediately after each fertilizer application during the 2012 season (and basal fertilizer application for winter wheat in 2011). Nutrient output comprises the nutrient content of the wheat grain, maize cobs and watermelon fruits produced on the fields.

The total nutrient output for each of the crops was calculated as the mass concentrations times the total wheat grain, or maize cobs, or watermelon fruit dry weight. Detailed information about nutrient output measurement can be found in S1 Text.

### Data analysis

Crop-specific yields under double cropping and intercropping are compared by using the partial land equivalent ratio (pLER). pLER is the ratio of the yield of a crop species in wheat-maize/watermelon intercropping, *Y*_I_, to the yield of sole species in wheat-maize double cropping or the water melon sole crop, *Y*_S_, i.e. pLER = *Y*_I_/*Y*_S_. If the pLER of a species is greater than its relative density, then the yield per plant is greater in the intercrop than in the sole crop [24].

Incomes obtained from the two systems are compared by calculating gross margins per ha. The gross margin is the difference between revenues and variable input costs, where revenues equal grain and fruit yield multiplied by grain and fruit price respectively. Grain and fruit yields were derived from field sampling; wheat, maize grain and fruit prices are local free market average prices obtained through inquiring of local dealers.

We estimated partial nutrient balances by comparing nutrient inputs of N, P, and K with nutrient removal from the field with the grain and fruit yield. Crop residues were returned to the field after harvest, therefore nutrients contained in the stover were not counted as a removal. Pairwise t-tests with uneven paired data were used to test differences in partial nutrient balances between the wheat-maize/watermelon system and the wheat-maize system in the year 2012 (using SPSS 20.0).

## Results

### Grain and fruit yields

Wheat-maize double cropping produced 14.8 ton grain per ha: 6.4 ton wheat grain per ha and 8.4 ton maize grain per ha, when averaged over the three growing seasons (Table 3). Average total grain yield in the wheat-maize/watermelon intercrop was 11.4 ton grain per ha: 4.7 ton wheat grain per ha and 6.7 ton maize grain per ha, representing yield reductions per ha of 26% and 19% in wheat and maize respectively. The average production of watermelon fruit in the intercrop ranged from 28.5 ton fresh fruit per ha in 2011 to 37.1 ton per ha in 2012 (Table 3).

**Table 3:** Grain and fruit yield (t ha^-1^) and partial Land Equivalent Ratios (pLER) of double cropping and intercropping systems.

Season	System	Sample size ^1^	Wheat		Watermelon		Maize	
			Grain yields ^3^	pLER_wheat_ ^4^	Fruit yields	pLER_watermelon_ ^5^	Grain yields	pLER_maize_
2010	Double cropping	9	5.3±1.4 ^2^		-		6.4±1.0	
	Intercropping	32	3.9±0.5	0.74	32.6±7.4	-	5.4±1.2	0.84
2011	Double cropping	10	7.0±0.8		-		7.9±0.4	
	Intercropping	34	5.1±0.5	0.73	28.5±7.8	-	6.9±0.8	0.88
2012	Double cropping	29	6.7±1.0		-		11.1±0.8	
	Intercropping	35	5.1±0.6	0.76	37.1±11.8	-	8.0±1.5	0.72
Mean ^6^				0.74 **		0.79		0.81 *

Source: Own crop yield monitoring data, 2010–2012

^1.^ Sample sizes differ between years (see S1 text for details).

^2.^ Values shown are means ± standard deviation.

^3.^ Grain yield is based on 13% moisture for wheat and 14% moisture for maize, while the yield of watermelon is fresh weight.

^4.^ pLER_wheat_, pLER_maize_ is the ratio of grain yield of respectively wheat or maize in the wheat-maize/watermelon system to that of the respective sole crop in wheat-maize double cropping.

^5.^ pLERwatermelon of watermelon is an approximate value based on intercropping watermelon yield divided by unpublished sole watermelon yield data (38.8 to 44.3 ton per ha).

^6.^* p<0.05, ** p<0.01, *** p<0.001; based on t-test of difference between the relative density of a crop and the pLER

The relative density of intercropped wheat under intercropping was 42% lower than under double cropping (see above) while its yield was 26% lower (pLER_wheat_ = 0.74; see Table 3) on average. Hence, the wheat yield per plant was 100×(74/58-1) = 28% larger in the intercropping system than in the double cropping system. For maize, the relative density of intercropped maize was 1.00 and its yield was 19% lower (pLER_maize_ = 0.81) under intercropping. Hence, maize yields per plant were 19% lower on average in the intercropping system, signaling competitive effects of watermelon on maize. Field trials (unpublished data) carried out in this area during 2012–2013 show that watermelon sole crop yields range from 38.8 to 44.3 ton per ha watermelon fresh weight. Based on this, we estimate the pLER_watermelon_ at approximately 0.79, indicating 21% lower yield per plant in the intercropping system.

### Economic performance

The average revenue per ha for the 2012 agricultural season was USD 9,732 in the wheat-maize/watermelon system and USD 6,069 in wheat-maize double cropping (Table 4). Slightly more than half (54%) of the revenue of the intercrop system was from watermelon (USD 5,286 per ha). Variable input costs were USD 3,037 per ha in the intercrop versus USD 1,701 per ha in the double cropping. As a result, the gross margin was equal to USD 6,695 in wheat-maize/watermelon, as compared to USD 4,367 per ha in double cropping. Thus, compared to double cropping, the integration of watermelon into the system increased revenues by 60%, variable costs by 79% and the gross margin by 53%. If an average farm of 0.58 ha would switch from wheat-maize double cropping to wheat-maize/watermelon intercropping on all its land, this would raise its total farm income from USD 2,534 to USD 3,883 on average.

**Table 4:** Revenues, variable costs, gross margins (USD ha^-1^) and labor use (hr ha^-1^) of the two cropping systems ^1^ ^,^ ^2^ in 2012.

	Wheat-maize double cropping (n = 29) ^3^			Wheat-maize/watermelon intercropping (n = 35)			
	Wheat	Maize	Total	Wheat	Maize	Watermelon	Total
Revenues ^4^	2,334±347	3,735±315	6,069±509	1,774±215	2,672±491	5,286±1,685	9,732±1,788
Seed/Seedling ^5^	99±19	118±15	217±26	48±42	134±21	213±114	394±121
Fertilizer	458±73	255±54	713±108	472±61	87±61	909±228	1467±253
Irrigation	219±64	8±12	227±63	183±67	1±2	265±60	449±97
Pesticides ^6^	37±19	41±24	78±32	36±13	22±10	186 ±87	245±94
Machinery ^7^	236±32	201±67	437±74	247±11	118±39	17±27	381±54
Mulching film ^8^	n/a	n/a	n/a	n/a	n/a	43±18	43±18
Fuel ^9^	11±7	19±8	30±11	9±8	16±9	32±15	57±26
Variable costs	1,059±119	642±87	1,701±151	994±129	379±86	1,664±315	3,037±373
Gross margin ^10^	1,275±348	3,093±331	4,367±550	780±226	2,293±481	3,622±1,542	6,695±1,612
Labor use (hr ha^-1^)	205±52	354±146	560±180	214±57	465±104	1,363±331	2,042±345
Gross margin per hour (USD hr^-1^)	6.22	8.74	7.82	3.64	4.93	2.66	3.28

Source: Own farm household survey, 2012

^1.^ All costs are converted into US Dollars based on the average exchange rate in 2012, 1 USD = 6.31 CNY.

^2.^ Values are shown as means ± standard deviation for the 59 farmers in the sample.

^3.^ n is the number of sampled fields.

^4.^ The prices of wheat, maize and watermelon in 2012 were 0.35, 0.33 and 0.14 US$/kg, respectively.

^5.^ Seed/seedling includes seeds of cereal crops and seeds or seedlings of watermelon.

^6.^ The category “pesticides” includes insecticides, fungicides, and herbicides.

^7.^ Machinery cost denotes the total expense for machinery rent, including the operator wage.

^8.^ Mulching film is only used in watermelon.

^9.^ Fuel includes diesel or gasoline consumed by farmers’ vehicles for transporting wheat and maize from the fields to home and watermelon to the market.

^10.^ Gross margin = revenues—variable costs.

Fertilizer was the largest cost item in both systems (Table 4): USD 1,467 per ha in wheat-maize/watermelon, versus USD 713 per ha in wheat-maize double cropping, representing an increase of 106%. Fertilizer costs made up 48% of the variable costs in wheat-maize/watermelon, and 42% in wheat-maize double cropping. The money spent on irrigation water was 98% higher in the intercropping system than in the wheat-maize double cropping. It accounts for 15% of the variable input costs in wheat-maize/watermelon and 13% in wheat-maize. Both fertilizer and water are used much more intensively in watermelon than in wheat and maize (Table 1), which explains their relatively high use in the intercropping system. Pesticides were a relatively minor costs item: 8% of the variable input costs both in wheat-maize/watermelon, and in wheat-maize. The extra pesticides expenditures in wheat-maize/watermelon cultivation were mostly made to control pests and diseases in watermelon.

Integration of watermelon into the wheat-maize system resulted in a major increase in labor use, from only 560 hours per ha in double cropping to as much as 2,042 hours per ha in intercropping, an increase of 265%. The increase in labor use was due to an increase in manual labor, as can be seen from Table 5. Pruning and harvesting of watermelon is not mechanized. Moreover, maize in the intercrop needs to be sown by hand to avoid damaging the watermelon plants. As a result, the use of hired machinery in 2012 was 13% lower in the intercropping system as compared to the double cropping system (Table 4).

**Table 5:** Time spent on cropping activities in 2012 (hr ha^-1^),

Agronomic practice/operation	Double cropping (n = 29) ^1^		Intercropping (n = 35)		
	Wheat	Maize	Wheat	Watermelon	Maize
Land preparation ^2^	0	0	0	103±78	0
Mulching ^3^	n/a	n/a	n/a	39±26	n/a
Sowing/Transplanting ^4^	0	0	0	135±48	153±51
Fertilization & Irrigation	94±42 ^8^	44±29	87±35	222±75	50±40
Spraying pesticides ^5^	25±12	17±13	28±16	110±71	17±14
Weeding ^6^	13±12	11±6	30±32	9±9	14±13
Pruning	-	-	-	431±173	-
Harvesting	73±24	283±125	68±27	287±121	224±95
Plant residue processing ^7^	0	0	0	27±39	7±22
Total per crop	205±52	354±146	214±57	1363±331	465±104
Total per system	560±180		2,042±345		

Source: Own farm household survey, 2012

^1.^ n is the number of interviewed farm households.

^2.^ Land preparation for wheat and maize planting is commonly conducted using machinery, but the preparation for watermelon is done by hand.

^3.^ Mulching film is only used in watermelon.

^4.^ All wheat is sown by sowing machines, so is maize in W-M system; maize in W-M/W system is sown by hand, while watermelon is transplanted manually.

^5.^ Pesticides includes insecticides and fungicides.

^6.^ Weeding includes killing weeds using herbicides and removal of weeds by hand.

^7.^ Plant residue processing indicates the time spent when watermelon roots are pulled out by hand and the time used for manually removing maize stalks from the field.

^8.^ Values are shown as means ± standard deviations.

Combining the gross margin and labor use estimates, we find that the gross margin per labor hour equaled USD 3.28 in the intercropping system versus USD 7.82 in the double cropping system. In other words, labor returns under the intercropping system were 58% lower than under the double cropping system.

### Nutrient budgets

The three macronutrients (N, P and K) showed substantial surpluses, both in wheat-maize and wheat-maize/watermelon, but more severely in the intercropping than in the double cropping system (Table 6). Inputs of N, P and K were 683 kg N, 273 kg P_2_O_5_ and 412 kg K_2_O per ha, respectively, in wheat-maize/watermelon, i.e. 76%, 41% and 155% higher than in wheat-maize double cropping (388 kg N, 194 kg P_2_O_5_ and 162 kg K_2_O per ha). Removals of N, P and K in maize-wheat/watermelon were 247 kg N, 128 kg P_2_O_5_ and 93 kg K_2_O per ha, and were -11%, 9% and 67% larger in intercropping than in double cropping (279 kg N ha^-1^, 117 kg P_2_O_5_ and 55 kg K_2_O per ha). As a result nutrient surpluses were much higher in intercropping than in double cropping, especially those of N and K. The surplus of N was 301% and the surplus of K was 200% higher in intercropping than in double cropping.

**Table 6:** Partial nutrient balances (kg ha^-1^) and fertilizer use efficiency of wheat-maize double cropping (n = 23) and wheat-maize/watermelon intercropping (n = 17).

Cropping System	Fertilizer input (kg ha^-1^)	Removal in product (kg ha^-1^)	Surplus (kg ha^-1^)	Fertilizer use efficiency ^1^ (%)
Nitrogen				
Double cropping	388±73 ^2^	279±31 **	109±77	72
Intercropping	683±142 *** ^3^	247±26	436±132 ***	36
Change	295	-32	327	36
Phosphorus (P_2_O_5_)				
Double cropping	194±42	117±15	77±44	60
Intercropping	273±47 ***	128±12 *	145±49 ***	47
Change	79	11	68	13
Potassium (K_2_O)				
Double cropping	162±50	55±6	106±51	34
Intercropping	412±108 ***	93±13 ***	320±104 ***	22
Change	251	37	213	12

Source: Own farm household survey and crop yield monitoring data, 2012

^1.^ Percentage of applied nutrients removed with product

^2.^ Values are shown as means ± standard deviations.

^3.^ * p < 0.05, ** p < 0.01, *** p < 0.001; based on t-tests of difference in mean between the two cropping systems.

## Discussion

Available evidence shows that intercropping sustains higher land productivity than cultivation of sole crops. It can also enhance and diversify farm incomes, particularly when a cash crop is integrated into an existing single or double grain cropping system. Limited research has been done so far on other economic aspects, like labor use, and the sustainability of such intercropping systems. The current study focuses on a novel intercropping system developed by farmers themselves. This system integrates a cash crop, watermelon, into the traditional wheat-maize double cropping system in the North China Plain. The main objective of our study is to obtain deeper insights into the economic performance and sustainability of this system taking into account prevailing resource scarcities and the economic and policy environment in the region.

Our analysis is based on crop yield monitoring data collected from 2010 to 2012 and data collected through a farm household survey held in 2012. By combining farm household survey data with on-field yield monitoring data we are able to make a profound analysis of the economic performance as well as the sustainability, as indicated in particular by the nutrient balances, of the intercropping system. In the farm household survey, we interviewed farmers younger than 70 years old with stated interests in agriculture and in improving their skills (see ‘S1 Text’). Our results should therefore not be interpreted as representative of all rural households living in the research area, but only of those households who obtain their incomes mainly from agriculture and who consider agriculture as their major livelihood strategy in the near future.

We find that labor use is more than three times larger in the intercropping system than in the traditional double cropping system. The cash crop (watermelon) that is introduced in the intercropping system requires much labor, while maize needs to be sown by hand in the intercropping system. So, although the revenues and gross margins of the intercropping system exceed those of the double cropping system by more than 50%, the returns per labor hour are much smaller under the intercropping system.

Fertilizer use is considerably higher in the intercropping system than in the traditional double cropping system. The nutrient balances that we calculated show substantial nutrient surpluses under both systems. But the estimated surpluses of N and K in the intercropping system were found to be around twice those under the double cropping system. In this section we discuss these findings against the background of the economic environment, food security and grain self-sufficiency policies, and natural resource degradation problems in the region.

### Economic environment

Cereal crop production in China and elsewhere generally provides limited income, but is also low risk because the price of cereals is relatively stable [25–27]. Cash crop production can be more lucrative but the profitability varies strongly with fluctuations in market prices. An intercropping system integrating a cash crop into cereals can therefore provide an attractive midway.

Intercropping wheat and maize with watermelon, as is done in some parts of the North China Plain, not only provides a profitable crop system innovation (Table 4), it intriguingly was attracting slightly younger farmers than the double cropping system (Table 2). Although we did not explicitly examine off-farm employment in our farm household survey, this finding suggests that at least some younger persons living in the region prefer working in the intercropping system over off-farm jobs. The integration of watermelon into the wheat-maize system resulted in a more than threefold increase in labor use but also in a decline in the gross margin per labor hour, from 7.82 USD in the double cropping system to 3.28 USD in the intercropping system (Table 4). These labor returns compare favorably with the average local agricultural wage (0.79 USD per hour) and non-agricultural wage (1.58 USD per hour) that we observed in the year 2012 in the research area. In other words, the intercropping system provided much more employment than the traditional double cropping system, and hence the need to work off-farm against relatively low wages during the lean season of the wheat-maize double cropping system was alleviated.

The price of watermelon shows considerable fluctuations over time. According to our on-farm interviews, prices ranged from 0.08 to 0.17 USD per kg over the last 10 years, but overall the price was rising. If the watermelon price would decline again to 0.08 USD per kg, the gross margin of the wheat-maize/watermelon system would be 4,430 USD per ha, similar to that of wheat-maize double cropping under current farm management practices. But the gross margin per labor hour would decline to around 2 USD per hour, only slightly exceeding the wage rate that can be earned elsewhere in the region.

### Food security and grain self-sufficiency policies

Generally, yields of component crops in polycultures are lower than those of sole crops because the former has lower planting density that the latter. In this study, the wheat-maize/watermelon intercropping produces a cash crop at the expense of grain yields. But it increases rural income by more than 50% and provides significantly more employment than the wheat/maize double cropping system. It can therefore play an important role in rural poverty alleviation and the improvement of household-level food security in the region.

Another major policy goal of the Chinese government, besides rural poverty alleviation, is to remain at least 95% self-sufficient in grain production. Polyculture systems may produce higher yields per unit planted area than sole cultures [12, 14, 28, 29], but the lower planting density of individual crops may contribute to lower output. In this study, averaged over all farms, wheat had a higher relative yield (pLER_wheat_ = 0.74) in the intercrop than its relative density (0.58). The larger wheat yield per plant under intercropping is the result of weak competition from watermelon because watermelon is planted later and not shading wheat [30, 31]. By contrast, maize had a lower relative yield (pLER_maize_ = 0.81) in the intercrop than its relative density (1.00). The lower yield per plant as compared to double cropping may be explained from two factors, i.e. the initial competition from earlier sown watermelon and postponed sowing dates of maize. In the intercropping system, maize is sown on fields with well-established watermelon plants that compete vigorously for light, water and nutrients. Farmers intentionally postpone maize planting in the intercrop because they assume this will reduce the negative impacts of maize on the fruit yield of watermelon, the most profitable crop in this intercropping system. However, the postponed sowing date reduces the number of growing days of maize, as the harvesting date is not delayed [32, 33], and increases the risk of yield loss due to unfertilized cobs.

The larger wheat yields and smaller maize yields per plant do not contribute to a larger overall grain output per ha. The area planted with wheat under intercropping is 42% smaller than under double cropping, while the area planted with maize is the same under the two systems. As a result, the average wheat output per ha in 2012 was 19% lower under intercropping, while the average maize output was 25% lower. Hence, the integration of watermelon into the traditional double cropping system on the North China Plain has negative effects for safeguarding grain self-sufficiency.

### Sustainable natural resource use

In the quest for high yields on scarce land resources, local farmers opt for over-use of nitrogen as they believe that higher yield demands high levels of nitrogen inputs [34] and that high watermelon quality requires high levels of potassium use [35]. The very large fertilizer use in the intercropping system (Table 6) is partly caused by the fact that wheat usually gets the same dose as is normally used for entire fields, while wheat is concentrated on the wheat strip with only 58% of the plants compared to the double cropping system. Such overuse causes nutrient surpluses and ensuing environmental problems [34, 36, 37], a phenomenon that is becoming an increasing serious environmental problem related to cash crop production in China [38]. Non-point source pollution caused by fertilizer and pesticide runoff from farmland is receiving more attention lately in China [39–42].

Our findings further show that almost twice as much irrigation water was consumed in 2012 by the intercropping system as compared to the double cropping system (Table 4). Water shortage has become a serious problem on the North China Plain in recent years [41, 43, 44]. Given that the consumption of water by industries and households is expected to increase rapidly in the near future, there is a strong need to reduce the use of irrigation water in Quzhou County and other parts of the North China Plain.

In conclusion, the intercropping system examined in this study will not be sustainable if the efficiency of nutrient and water use is not significantly improved. Nutrient management of cereal crops has been intensively studied [34, 36, 37, 45, 46], but little is known so far about how to improve nutrient (and water) management of intercropping systems.

## Conclusion

Our study indicates that intercropping cereals with a cash crop like watermelon may not only contribute to higher agricultural incomes, but also become an important source of rural employment provided the incomes earned per hour in the intercropping system exceed the wages that can be earned off-farm. The recent increases in migrant wages paid in China’s urban areas pose a major challenge in this respect.

There is an urgent need for more research on improving the wheat-maize/watermelon and similar intercropping systems by adjusting sowing dates and optimizing nutrient and water input use. The development of improved intercropping systems will not only contribute to the economic viability of these systems under rising wage levels, but also reduce their negative effects on grain self-sufficiency and natural resource degradation.

Additionally, more research is needed on the age and gender composition of laborers working in intercropping systems like the system that we analyze in this paper, as compared to the traditional wheat-maize double-cropping system in the North China Plain. Given that women and children of school-going age normally need to spend a considerable amount of their time on non-agricultural tasks as well, the additional employment generated by the intercropping system may have undesirable side-effects that we did not address in this study.

## Supporting Information

S1 Table Questionnaire of household survey in 2012. (DOC)

S2 Table Fertilization survey after each application. (DOC)

S1 Text Methods used for plot sampling and yield monitoring. (DOCX)
