# Supplementary material for: Economic Performance and Sustainability of a Novel Intercropping System on the North China Plain
Source: PLoS One. 2015 Aug 14;10(8):e0135518. doi: 10.1371/journal.pone.0135518 (PMC4537243; doi:10.1371/journal.pone.0135518)
Supplement: S1 Table — (DOC) [file pone.0135518.s001.doc]

**Supporting Information**

**S2 Table. Questionnaire of household survey in 2012**

This questionnaire was designed for a larger number of studies. Only those parts of the survey that were used for the current study are presented.

| **Cropping system:**  **Farmer code：** | **SC0**  (1=wheat-maize, 2=wheat-maize/watermelon)  **SC1** |
| --- | --- |
| **Township:** | **SC2** |
| **Village:** | **SC3** |
| **Name of Householder:** | **SC4** |
| **Name of Respondents:** | **SC5** |
| [**Telephone**](javascript:void(0);) **Number:** | **SC6** |
| **Mobile Phone:** | **SC7** |
| **Survey Date:** | **SC8** |
| **Name of Investigator:** | **SC9** |

1. **Basic information of family members**

**Labor population (16-70 years old) of your family A1 (number) _______ , No. of family members A2(number)_____ .**

| Member code | Relationship with Householder | | Gender | Age | [Education](javascript:void(0);) [Years](javascript:void(0);) | Labor Force^*^ | Village Cadre |
| --- | --- | --- | --- | --- | --- | --- | --- |
| Unit of Code | Name | Code 1 | 1=male, 2=female | Years old | Year | 1=Yes, 0=No | 1=Yes, 0=No |
| **Pid** | **A3** | **A4** | **A5** | **A6** | **A7** | **A8** | **A9** |
| 1 |  |  |  |  |  |  |  |
| 2 |  |  |  |  |  |  |  |
| 3 |  |  |  |  |  |  |  |
| 4 |  |  |  |  |  |  |  |
| 5 |  |  |  |  |  |  |  |
| 6 |  |  |  |  |  |  |  |

Code 1 (relationship with householder): 1=him/herself; 2=spouse; 3=son/daughter; 4=grandson/granddaughter; 5=father/mother; 6=brother/sister; 7=son /daughter-in-law; 8=brother /sister-in-law; 9=parents-in-law; 10=other relative; 11= no relation.

(*Labor force indicates whether the interviewee really works on agricultural field)

1. **Arable land use of each household in 2012 (1 mu = 667 m^2^)**

|  |  | | **Land areas of various cropping systems adopted by local farmers** | | | | | | |
| --- | --- | --- | --- | --- | --- | --- | --- | --- | --- |
| item | Total area of arable land | Number of farmland | Wheat-maize double cropping | Wheat-maize/watermelon intercropping | Wheat/watermelon-vegetables intercropping | Watermelon/cotton intercropping | Sole cotton | Other cash crops | other crops |
| unit | mu | no. | mu | mu | mu | mu | mu | mu | mu |
| variable | **B1** | **B2** | **B3** | **B4** | **B5** | **B6** | **B7** | **B8** | **B9** |
| Survey |  |  |  |  |  |  |  |  |  |

**Table C:** Not used in this study

**D1. Winter wheat-Input of Agricultural Production and Labor Force** (Land area: ______mu, Seeding rate: _____kg/mu; Seed price:____ yuan/kg)

| Item | Unit | Variable | Survey |
| --- | --- | --- | --- |
| Whether **subsoiling** before wheat sowing in 2011? | 1=Yes; 0=No -> D03 | D01 |  |
| Price of **subsoiling** (including cost of own fuel) | yuan (No = 0 if no subsoiling) | D02 |  |
| Whether [**rotary**](javascript:void(0);) [**tillage**](javascript:void(0);) before wheat sowing in 2011? | 1=Yes; 0=No -> D05 | D03 |  |
| Price of [**rotary**](javascript:void(0);) [**tillage**](javascript:void(0);) (including cost of own fuel) | yuan (No = 0 if no tillage) | D04 |  |
| Time consumed for **land preparation before wheat sowing** | hr | D05 |  |
| Whether **hiring labor for land preparation**? | 1=Yes; 0=No -> D08 | D06 |  |
| Price of **hiring labor** | yuan | D07 |  |
| Methods of **applying basic fertilizer** | 1=broadcast, 2=furrow, 3=other | D08 |  |
| Time used for **basic fertilizer application** | hr | D09 |  |
| Whether applying **manure** for wheat？ | 1=Yes, 0=No | D10 |  |
| Time used for **manure application** in wheat | hr | D11 |  |
| Price of **manure** | yuan | D12 |  |
| Price of **sowing wheat** (including cost of own fuel) | yuan | D13 |  |
| Whether **performing** **Irrigation before overwintering** (including irrigation before sowing/ emergence and winter water)？ | 1=Yes; 0=No -> D14 | D14 |  |
| Price of **irrigation** | yuan/mu | D15 |  |
| Time consumed for **irrigation** | hr (Note: multiplication by no. of participant labor) | D16 |  |
| Method of **weeding** | 1=herbicide, 2=manpower, 3=other | D17 |  |
| Cost of **weeding** | yuan (manpower = 0) | D18 |  |
| Time consumed for **weeding** | hr | D19 |  |
| Whether **loosening topsoil** using rake？ | 1=Yes; 0=No -> D20 | D20 |  |
| Cost for **loosening topsoil** | yuan | D21 |  |
| Time consumed for **loosening topsoil** | hr | D22 |  |
| Way of **1^st^ irrigation** after regreening | 0=No, 1=flooding, 2=furrow, 3=other | D23 |  |
| Time consumed for **1^st^ irrigation** after regreening | hr | D24 |  |
| Cost of **1^st^ irrigation** after regreening | yuan/mu | D25 |  |
| Way of **2^nd^ irrigation** after regreening | 0=No, 1=flooding, 2=furrow, 3=other | D26 |  |
| Time consumed for **2^nd^ irrigation** after regreening | hr | D27 |  |
| Cost of **2^nd^ irrigation** after regreening | yuan/mu | D28 |  |
| Way of **3^rd^ irrigation** after regreening | 0=No, 1=flooding, 2=furrow, 3=other | D29 |  |
| Time consumed for **3^rd^ irrigation** after regreening | hr | D30 |  |
| Cost of **3^rd^ irrigation** after regreening | yuan/mu | D31 |  |
| Number of times for **spraying insecticide** | no. of per piece of land | D32 |  |
| Time consumed for **spraying insecticide** | hr each time | D33 |  |
| Cost of **spraying insecticide of wheat** | yuan each time | D34 |  |
| Way of **harvesting wheat** | 1=machinery, 2=manpower, 3=other | D35 |  |
| Time consumed for **harvesting wheat** | hr | D36 |  |
| Cost of **harvesting wheat** | yuan/mu | D37 |  |
| Cost of **hiring labor for harvesting wheat grain** | yuan（if not hiring, cost=0） | D38 |  |
| Time consumed for **drying and storing up wheat grain** | hr | D39 |  |
| Fuel cost of **transporting fertilizer, wheat grain and so on** | yuan | D40 |  |
| Whether **returning** **wheat straw** to field? | 1=Yes, 0=No | D41 |  |
| Time consumed for **straw returning** | hr | D42 |  |
| Cost of **straw returning** | yuan | D43 |  |

**D2. Maize-Input of Agricultural Production and Labor Force** (Land area: ______mu )

| Item | Unit | Variable | Option |
| --- | --- | --- | --- |
| Whether **preparing land for maize sowing**? | 1=Yes, 0=No -> D54 | D51 |  |
| Time consumed for **land preparation** | hr | D52 |  |
| Cost of **land preparation** | yuan | D53 |  |
| Way of **sowing maize** | 1=machinery, 2=manpower, 3=other | D54 |  |
| Price of **maize seed** | yuan/bag or yuan/500g | D55 |  |
| **Seeding rate** of maize | bag/500g per piece of land | D56 |  |
| Time consumed for **sowing maize** | hr | D57 |  |
| Price of sowing maize | yuan/mu | D58 |  |
| Whether **performing** **irrigation before or after sowing maize**? | 1=Yes, 0=No | D59 |  |
| Time consumed for **irrigation** | hr | D60 |  |
| Price of **irrigation** | yuan/mu | D61 |  |
| Whether **thinning or transplanting seedlings** after maize emergence? | 1=Yes, 0=No -> D65 | D62 |  |
| Time consumed for **thinning or transplanting** | hr | D63 |  |
| Cost of **thinning or transplanting** | yuan | D64 |  |
| Whether **topdressing** to maize? | 1=Yes, 0=No -> D68 | D65 |  |
| Way of **topdressing** | 1=furrow, 2=application with water, 3=hole application, 4=other | D66 |  |
| Time consumed for **topdressing** | hr | D67 |  |
| Whether **performing irrigation** after topdressing? | 1=Yes, 0=No -> D71 | D68 |  |
| Time consumed for **irrigation** | hr | D69 |  |
| Price of **irrigation** | yuan/mu | D70 |  |
| Time consumed for **weeding** | hr | D71 |  |
| Cost of **weeding** | yuan | D72 |  |
| Whether **applying chemicals for preventing lodging**？ | 1=Yes, 0=No -> D76 | D73 |  |
| Time consumed for **chemical control** | hr | D74 |  |
| Cost of **chemical control** | yuan | D75 |  |
| Number of times for **spraying insecticide** | no. of per piece of land | D76 |  |
| Time consumed for **spraying insecticide** | hr each time | D77 |  |
| Cost of **spraying insecticide** | yuan each time | D78 |  |
| Way of **harvesting maize** | 1=machinery, 2=manpower, 3=other | D79 |  |
| Time consumed for **harvesting maize** | hr | D80 |  |
| Cost of **harvesting maize** | yuan/mu | D81 |  |
| Whether **threshing** maize grain pre-selling? | 1=Yes, 0=No -> D85 | D82 |  |
| Time consumed for **threshing** | hr | D83 |  |
| Cost of **threshing** | yuan | D84 |  |
| Whether **returning maize straw** to field? | 1=Yes, 0=No -> D88 | D85 |  |
| Time consumed for **straw returning** | hr | D86 |  |
| Price of **straw returning** | yuan/mu | D87 |  |
| Fuel cost for **transporting fertilizer, maize ear and so on** | yuan | D88 |  |
| Whether **hiring labor** during maize growing season | 1=Yes, 0=No | D89 |  |
| Cost of **hiring labor** | yuan | D90 |  |

**E. Watermelon-Input of Agricultural Production and Labor Force** (Land area: ______mu)

| Item | Unit | Variable | Option |
| --- | --- | --- | --- |
| Whether **using** **manure**? | 1=Yes, 0=No -> E05 | E01 |  |
| Total amount of **manure application** | m^3^ | E02 |  |
| Price of **manure** | yuan/m^3^ | E03 |  |
| Time consumed for **manure application** | hr | E04 |  |
| Way of **land preparation** | 1=machinery, 2=manpower, 3=other | E05 |  |
| Time consumed for **land preparation** | hr | E06 |  |
| Price of **land preparation** | yuan/mu | E07 |  |
| Time consumed for **irrigation after land preparation** | hr | E08 |  |
| Cost of **irrigation** | yuan/mu | E09 |  |
| Time consumed for **weeding** | hr | E10 |  |
| Cost of **weeding** | yuan | E11 |  |
| Time consumed for **filming mulch** | hr | E12 |  |
| Cost of **filming mulch** | yuan | E13 |  |
| Price of **watermelon seed** | yuan/box | E14 |  |
| **Sowing amount** of watermelon | box | E15 |  |
| Time consumed for **sowing** | hr | E16 |  |
| Time consumed for **filling/transplanting seedlings** | hr | E17 |  |
| Whether using **graft watermelon seedling**? | 1=Yes, 0=No -> E23 | E18 |  |
| Price of **graft watermelon seedling** | yuan/plant | E19 |  |
| **Transplanting amount** of graft seedling | plant | E20 |  |
| Time consumed for **transplanting graft seedling** | hr | E21 |  |
| Time consumed for **filling graft seedling** | hr | E22 |  |
| Time consumed for **clearing up** [**tendrils**](javascript:void(0);) **before wheat harvest** | hr | E23 |  |
| Time consumed for **clearing up** [**tendrils**](javascript:void(0);) **after wheat harvest** | hr | E24 |  |
| Way of **1^st^ topdressing** | 1=hole, 2=furrow, 3=application with water, 4=other | E25 |  |
| Time consumed for **1^st^ topdressing** | hr | E26 |  |
| Cost of **1^st^ irrigation** | yuan/mu | E27 |  |
| Way of **2^nd^ topdressing** | 1=hole, 2=furrow, 3=application with water, 4=other | E28 |  |
| Time consumed for **2^nd^ topdressing** | hr | E29 |  |
| Cost of **2^nd^ irrigation** | yuan/mu | E30 |  |
| Way of **3^rd^ topdressing** | 1=hole, 2=furrow, 3=application with water, 4=other | E31 |  |
| Time consumed for **3^rd^ topdressing** | hr | E32 |  |
| Cost of **3^rd^ irrigation** | yuan/mu | E33 |  |
| Times of **fruit thinning** | no. of piece of land | E34 |  |
| Time consumed for **fruit thinning** | hr/mu each time | E35 |  |
| Number of times for **spraying insecticide** | no. of per piece of land | E36 |  |
| Time consumed for **spraying insecticide** | hr each time | E37 |  |
| Cost of **spraying insecticide** | yuan each time | E38 |  |
| Time consumed for **harvesting watermelon fruit** | hr | E39 |  |
| Time consumed for **straw processing** | hr | E40 |  |
| Fuel cost for **transporting fertilizer, fruit and so on** | yuan | E41 |  |
| Whether **hiring labor** during watermelon growing season | 1=Yes, 0=No | E42 |  |
| Cost of **hiring labor** | yuan | E43 |  |
| Whether **using insecticide** before land ploughing? | 1=Yes, 0=No | E44 |  |
| Cost of **insecticide** before land ploughing? | yuan | E45 |  |
