# Supplementary material for: Economic Performance and Sustainability of a Novel Intercropping System on the North China Plain
Source: PLoS One. 2015 Aug 14;10(8):e0135518. doi: 10.1371/journal.pone.0135518 (PMC4537243; doi:10.1371/journal.pone.0135518)
Supplement: S2 Table — (DOC) [file pone.0135518.s002.doc]

**Supporting Information**

**S3 Table. Fertilization survey after each** application

| Fertilization Survey during 2011-2012 growing season (crop and crop stage) | | | | | | | | | | | |
| --- | --- | --- | --- | --- | --- | --- | --- | --- | --- | --- | --- |
| Investigator: (full name) ; Date: yyyy-mm-dd; | | | | | | | | | | | |
| name | code | land area  (mu^*^) | fertilizer | | | | | | | | |
|  |  |  | date | way | fertilizer type | kg per bag or m^3^ | yuan per bag | application amount  (kg) | N% | P_2_O_5_% | K_2_O% |
|  |  |  |  |  |  |  |  |  |  |  |  |
|  |  |  |  |  |  |  |  |  |  |  |  |
|  |  |  |  |  |  |  |  |  |  |  |  |
|  |  |  |  |  |  |  |  |  |  |  |  |
|  |  |  |  |  |  |  |  |  |  |  |  |
|  |  |  |  |  |  |  |  |  |  |  |  |
|  |  |  |  |  |  |  |  |  |  |  |  |
|  |  |  |  |  |  |  |  |  |  |  |  |
|  |  |  |  |  |  |  |  |  |  |  |  |

Code of fertilization way: 1=broadcast, 2= furrow, 3= application with water, 4=other.

Code of fertilizer type: 1= straight fertilizer; 2= compound fertilizer; 3=manure and manure type (cow, pig and so on); 4=other.

*mu is the unit commonly used by farmers, 1 mu = 667 m^2^.
