# Supplementary material for: Economic Performance and Sustainability of a Novel Intercropping System on the North China Plain
Source: PLoS One. 2015 Aug 14;10(8):e0135518. doi: 10.1371/journal.pone.0135518 (PMC4537243; doi:10.1371/journal.pone.0135518)
Supplement: S1 Text — (DOCX) [file pone.0135518.s003.docx]

**Supporting Information**

**S1 Text. Methods used for plot sampling and yield monitoring**

The study village, Houlaoying, had 265, 332 and 375 households in 2010, 2011 and 2012 respectively. Households are living in six natural villages (village groups), each with its own group leader. The village territory includes 156 ha arable land, of which about one half was used for wheat-maize double cropping in 2010, more than 25% for wheat-maize/watermelon and the remaining for other cropping systems. No large differences were observed between years. The average area of plots grown to major crops is about 0.2 ha. Wheat-maize/watermelon intercropping was found mainly in an area covering roughly 40% of the village territory with good access to irrigation and roads to transport harvested products, hereafter area 1. In the remaining 60% of the village territory (hereafter area 2) intercropped fields were few and scattered.

The following three steps were taken to select candidate farm households for the household survey and for yield monitoring that would yield farm households interested in longer term engagement in agriculture and innovative cropping systems. Firstly, we asked the six village group leaders which cropping systems were used by the households in their natural village. Households in two village groups mainly grew fruit trees, cotton and vegetables. These two were therefore dropped. Secondly, in the remaining four village groups we selected farmers younger than 70 years old because persons above that age are usually no longer involved in farming. The proportion of persons aged over 70 is less than 5% in Houlaoying village. Thirdly, we talked personally with the remaining farmers if they were interesting in agricultural practices and improving their skills, and if they were willing to participate in this study about “old” practices and “new” practices that they had developed. The farmers who responded positively to these questions were considered potential interviewees.

Hereafter we sampled fields in another three steps. In area 1, one block of fields was selected for each of the four village groups. Within a block, fields were excluded from the sample if the owner was not among the selected farm households, or if the field was not cropped to one the two cropping systems in this study. In this way a total of 38, 50 and 50 fields were sampled in 2010, 2011 and 2012 respectively. In area 2 one sampling point was randomly selected from each of the blocks cultivated by farmers in each of the four village groups, and a few fields (3, 5 and 5 in 2010, 2011 and 2012, respectively) in the vicinity of the sampling points were selected if cultivated by a farmer from our list of preselected interviewees and if cultivated to one of the selected cropping systems.

At the start of the cropping years 2010, 2011 and 2012, this sampling procedure resulted in 41, 58 and 35 intercropped fields and 9, 17 and 29 double cropped fields, respectively, that were used for yield monitoring. In 2010 and 2011 a relatively large number of intercropped fields was selected as we assumed that variation in intercropping practices between farmers would be larger than for double cropping, because intercrops are mostly cultivated by hand while double cropping is highly mechanized. In 2012 we increased the sample size of double cropping. As most farmers continued practicing double and intercropping on the same plots in subsequent years, 80% of the fields used in 2010 were also sampled in 2011 and 2012. To maintain sample size, some new fields located in the vicinity of the fields sampled earlier were added in 2011 and 2012, and the households managing these plots were included in the 2012 household survey. Unfortunately, some of the selected fields had to be dropped from our sample as some farmers harvested their crops before samples were taken and a few other farmers changed their original cropping plans. As a result, the number of plots used for yield monitoring varies between years and between cropping systems (see Table 3).

Measurements of grain yield were made at crop maturity. In each sampled wheat and maize field three subsamples were taken. A subsample in wheat consisted of a one meter-long strip of seven adjacent rows. Harvested plants were threshed and the fresh grains weighed. A subsample in maize consisted of a five meter-long strip of four maize rows. All maize cobs were threshed manually after air drying and the fresh grains were weighed. From each plot subsamples were bulked and a 500-g sample of wheat or maize grain was dried to constant weight in an oven at 70 °C, to determine water content of fresh grain. The reported final yields were standardized to 13% moisture for wheat and 14% moisture for maize. Watermelon fruits were picked when mature from late July to mid-August and sold in the free market. The farmers whose fields were used for the study recorded the fresh weight of watermelons collected from the field. The fresh weight was determined using a wagon balance (2 kg to 200 ton operating range). At the end of the watermelon season, the total fresh fruit yield for each plot was calculated.

For wheat nutrients, dry samples to determine mass concentration of grains were taken from the wheat grain subsamples that were used for determining water content. For maize, the cobs of 10 maize plants were threshed and both core and grains were oven-dried at 70°C, weighed and then ground for chemical analysis. For watermelon, the fruits of 6 representative plants were collected. A 200-g slice of each fruit was taken and the bulked sample was oven-dried at 70°C for 5 to 7 days to constant weight, weighed and then ground for chemical analysis.

The ground dried samples were wet-digested with concentrated H_2_SO_4_ and H_2_O_2_ (30% v/v), and then nitrogen content was determined by the micro-Kjeldahl procedure, phosphorus content by the vanadomolybdate method, and potassium content by flame photometry (FP640, Shanghai Instrument Co. Ltd., Shanghai, China) [1].

**Reference**

1. Horwitz W. Official methods of analysis: AOAC Washington, DC; 1980.
